# Effectiveness, immunogenicity and safety of 23-valent pneumococcal polysaccharide vaccine revaccinations in the elderly: a systematic review

**DOI:** 10.1186/s12879-016-2040-y

**Published:** 2016-11-25

**Authors:** Cornelius Remschmidt, Thomas Harder, Ole Wichmann, Christian Bogdan, Gerhard Falkenhorst

**Affiliations:** 1 Robert Koch Institute, Immunization Unit, Seestrasse 10, 13353 Berlin, Germany; 2 Mikrobiologisches Institut–Klinische Mikrobiologie, Immunologie und Hygiene, Friedrich Alexander Universität (FAU) Erlangen-Nürnberg and Universitätsklinikum Erlangen, 91054 Erlangen, Germany

## Abstract

**Background:**

In many industrialized countries routine vaccination with the 23-valent pneumococcal polysaccharide vaccine (PPSV-23) is recommended to prevent pneumococcal disease in the elderly. However, vaccine-induced immunity wanes after a few years, and there are controversies around revaccination with PPSV-23. Here, we systematically assessed the effectiveness and safety of PPSV-23 revaccination.

**Method:**

We conducted a systematic literature review in MEDLINE, EMBASE, and Cochrane Central Register of Controlled Trials from inception to June 2015. We included all study types that compared effectiveness, immunogenicity and/or safety of PPSV-23 as a primary vs. a revaccination dose in persons aged 50 years and older. With respect to immunogenicity, we calculated the ratio of geometric mean antibody concentrations and opsonophagocytic indexes at identical time-points after primary and revaccination. Additionally, we compared rates and severity of adverse events (AEs) after primary and revaccination.

**Results:**

We included 14 observational studies. 10 studies had a prospective design and analysed data on (i) the same individuals after a first and a second dose of PPSV-23 given 1 to 10 years later (*n* = 5) or (ii) two groups consisting of participants receiving PPSV-23 who were either vaccine-naïve or had received a first PPSV-23 dose 3 to 13 years earlier (*n* = 5). Three studies used electronic data bases to compare AEs after primary vs. revaccination doses of PPSV-23 after 1 to 10 years and one study had a cross-sectional design. Number of participants in the non-register-based and register-based studies ranged from 29 to 1414 and 360 to 316,000, respectively. 11 out of 14 included studies were at high risk of bias, three studies had an unclear risk of bias. None of the studies reported data on clinical effectiveness. Immunogenicity studies revealed that during the first two months antibody levels tended to be lower after revaccination as compared to primary vaccination. Thereafter, no obvious differences in antibody levels were observed. Compared to primary vaccination, revaccination was associated with an increased risk of local and systemic AEs, which, however, were usually mild and self-limiting. The risk and severity of AEs appeared to decrease with longer intervals between primary and revaccination.

**Conclusion:**

Data comparing the effectiveness of primary vs. revaccination with PPSV-23 are still lacking, because there are no studies with clinical endpoints. Data from observational studies indicates that revaccination with PPSV-23 is likely to induce long-term antibody levels that are comparable to those after primary vaccination. Given the high disease burden and the waning of vaccine-induced immunity, revaccination with PPSV-23 could be considered in the elderly. The increased risk of local and systemic AEs can likely be mitigated when giving revaccination at least five years after the primary dose. Adequately powered randomized controlled trials using clinical endpoints are urgently needed.

**Electronic supplementary material:**

The online version of this article (doi:10.1186/s12879-016-2040-y) contains supplementary material, which is available to authorized users.

## Background

Pneumococcal disease is a major cause of morbidity and mortality in the elderly population worldwide [1–3]. Therefore, National Immunization Technical Advisory Groups (NITAGs) in most industrialized countries recommend vaccination of the elderly against pneumococcal disease [4–6]. There are currently two different pneumococcal vaccines approved for the use in the elderly: a polysaccharide vaccine containing 23 different capsular polysaccharides (serotypes) of *Streptococcus pneumoniae* (PPSV-23) and a 13-valent conjugate vaccine (PCV-13).

PPSV-23 has been available since 1983. There is evidence that its protective effect declines already 3 to 5 years after vaccination [7, 8]. At the same time pneumococcal disease incidence among the elderly increases with age, which calls for PPSV-23 revaccination [9]. However, it has been postulated that repeat vaccination leads to hypo-responsiveness resulting in diminished antibody response [10]. Moreover, conflicting reports exist regarding an increased risk of adverse events (AE) following revaccination, as compared to the primary vaccine dose [11–14].

After licensure of PCV-13 had been extended for adults in 2011, many NITAGs continue to recommend PPSV-23 for elderly [4, 5, 15, 16]. Due to significant herd protection effects induced by routine childhood vaccination with PCV-13, those additional 11 serotypes in PPSV-23, which are not covered by PCV-13, are gaining epidemiological importance also among the elderly [17–19]. Therefore, the questions related to PPSV-23 revaccination continue to be of high relevance, both for clinicians but also for many NITAGs that are in the process of updating their guidelines on adult pneumococcal vaccination in view of new evidence and the licensure of PCV-13 for adults.

The purpose of this review was therefore to systematically assess differences in the effectiveness, immunogenicity and safety of revaccination as compared to primary vaccination with PPSV-23 in the elderly population.

## Methods

### PRISMA-guideline and study protocol

The systematic review was performed according to the Preferred Reporting Items for Systematic Reviews and Meta-analyses (PRISMA) statement [20] and was prospectively registered with the international prospective register of systematic reviews (PROSPERO) (Reg. no. CRD42015024145).

### Eligibility criteria

We evaluated all original studies that reported effectiveness, immunogenicity or safety of revaccination with PPSV-23 as compared to a primary PPSV-23 dose in persons aged 50 years or older, irrespective of underlying comorbidities. No restrictions were made regarding publication language and publication status (published/unpublished studies). We excluded studies in which other vaccine types were used (e.g., experimental vaccines; pneumococcal polysaccharide vaccines with less than 23 serotypes [e.g., PPSV-14]; pneumococcal conjugate vaccines) or if a revaccination dose was administered within 1 year of the first PPSV-23 dose.

### Outcomes

Regarding vaccine effectiveness, we considered all clinical outcomes including invasive pneumococcal disease, pneumonia (vaccine-type and all-types), as well as non-specific outcomes such as all-cause mortality or hospitalization. Immunogenicity data were considered, if geometric mean antibody concentrations (GMCs) or opsonophagocytic indexes (OPIs) were measured at the same interval after primary vaccination and revaccination dose (e.g., 1 month after primary and 1 month after revaccination), respectively. Since seroconversion is difficult to define in the absence of a validated protective threshold, we evaluated absolute antibody levels (GMC or OPA titers) rather than seroconversion rates. Regarding safety, we considered all types of local and systemic AE that were reported in the included studies.

### Literature search

Electronic databases searched were MEDLINE, EMBASE, and Cochrane Central Register of Controlled Trials (date of last search 26.06.2015). The search strategy included both keywords and MESH terms related to effectiveness, immunogenicity or safety of PPSV-23. For complete search strategy, see Additional file 1. In addition, we manually searched reference lists of all relevant original studies and reviews and searched ClinicalTrials.gov for additional studies.

### Literature screening and data extraction

Two reviewers (CR, TH) independently screened titles, abstracts and full text articles. From eligible studies, two reviewers (CR and GF) extracted study data and assessed methodological quality, using standardized extraction forms. The extraction forms were pilot tested with the first two identified studies. After calibration of the extraction process, CR and GF finally extracted the following information: author, publication year, country, study design, study population, age of participants, study size, number of PPSV-23 doses administered, time span between first and revaccination doses, outcome measures, laboratory methods, serotypes measured, confounders considered in the statistical analysis and potential conflicts of interests of the authors. In case of disagreements regarding the screening process, data extraction, and quality assessment a final decision was made by consensus or resolved by a third reviewer (GF [literature screening] or TH [data extraction process]).

### Risk of bias assessment

We used the Cochrane risk of bias tool to assess risk of bias for RCTs [21] and the Critical Appraisal Skills Program (CASP) tool for observational studies (http://www.casp-uk.net/checklists). According to the suggestions of the Cochrane Collaboration, we gave particular attention to the domains risk of selection bias, detection bias and attrition bias in observational studies. For each study, risk of bias appraisal was expressed as considered judgment as either “low”, “high” or “unclear”.

### Statistical analysis

Abstracted data were aggregated in tables. Risk ratios (RR), odds ratios (OR) and corresponding 95% confidence intervals (95% CIs) were either calculated or directly extracted from the publications, if available. To assess differences in serotype-specific GMCs after the primary and revaccination dose, we graphically displayed numbers with 95% CI for the most commonly analyzed serotypes 4, 6B, 14 and 23 F. We also calculated GMC (and OPI) ratios (i.e., GMC (OPI) after revaccination/GMC (OPI) after primary vaccination) with corresponding 95% CIs at each available time point. A ratio of > 1 indicates a higher antibody level after revaccination than after primary vaccination. A ratio of < 1 indicates a lower antibody level after revaccination than after primary vaccination. Ratios with 95% CIs were calculated by back transforming the mean difference GMCs between the vaccination groups on the logarithmic scale [22].

Ratios are displayed separately for each study and for the most commonly analyzed serotypes. Due to marked differences within studies (immunogenicity differences between serotypes) and between studies (e.g., different study populations, different time spans from primary vaccination to revaccination, different laboratory methods), we did not perform meta-analyses. Calculations were performed using STATA 12 (StataCorp LP, Texas, USA).

## Results

**Fig. 1:**
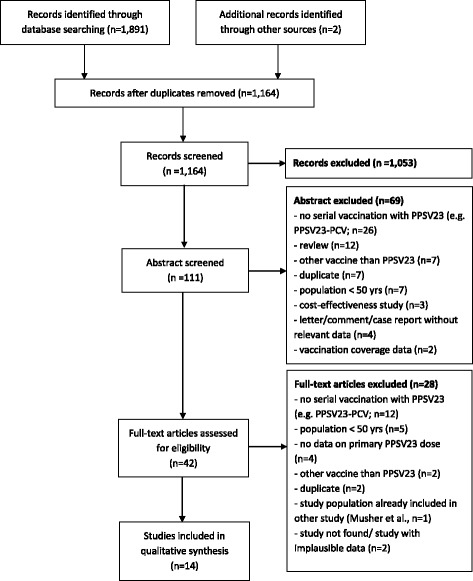
Flow chart for the systematic literature search and study selection

The literature search yielded a total of 1162 titles and two additional studies were identified through other sources (Clinicaltrials.gov; reference list of an identified review) (Fig. 1). Finally, 14 studies fulfilled the eligibility criteria [11–13, 22–32]. For excluded studies see Additional file 1.

### Characteristics of included studies

Of the 14 studies, two studies were randomized controlled trials (RCTs) [23, 31]. However, since only data from one study arm were relevant for our research question, we treated both trials as cohort studies. The remaining studies were prospective (*n* = 8) or retrospective (*n* = 3) cohort studies, and one study had a cross-sectional design (for study characteristics see Table 1). The majority of studies comprised resident elderly populations (*n* = 10), the remaining included only patients with chronic obstructive pulmonary disease [23, 29], patients with a history of community-acquired pneumonia [24] or renal transplant recipients [31]. Five studies had a longitudinal design in which the same individuals received a primary dose and a revaccination with PPSV-23 one to ten years later [22, 24, 28, 29, 31].

**Table 1:** Characteristics of included studies

Author, year [Ref]	Country	Study population^*1^	Participants (n) receiving primary/revaccination dose	Time span between primary and revaccination	Serotypes measured (laboratory methods)	Funding
Prospective cohort studies (*n* = 10)						
Tobudic, 2012^2^ [31]	Austria	Adult renal transplant recipients, 50.5 (±13) yrs	29 (longitudinal cohort)	1 year	1, 4, 5, 7F, 9V, 14, 18C, 19F, 23F (WHO-ELISA)	Oesterreichische Nationalbank
Dransfield, 2012^2^ [23]	USA	Patients with COPD, 64 (±10) yrs	42/48	Mean 8.4 (±3.5) years	4, 6B, 9V, 14, 18C, 19F, 23F (WHO-ELISA; OPA)	National Heart, Lung, and Blood Institute
Hammitt, 2011 [11]	USA	Alaska native population, 55–74 years	123/121 (2nd dose) and 71 (3rd or 4th dose)	6–22 years	1, 4, 6B, 14, 19 F (non- WHO-ELISA; OPA)	US Department of Health and Human Services - National Vaccine Program Office
Jackson, 1999 [13]	USA	Resident population, 50–74 years	901/513	5–13 (median 6) years	4, 14, 23F (non-WHO-ELISA; OPA)	CDC and Lederle Laboratories
Jackson, 2013 [22]	USA	Resident population with stable underlying chronic conditions, 60–64 years	157–181 (longitudinal cohort)^3^	3.5–4 years	1, 3, 4, 5, 6A, 6B, 7F, 9V, 14, 18C, 19A, 19F, 23F (OPA)	Wyeth Vaccine Research/Pfizer
Manoff, 2010^4^ [25]	USA	Resident population, 65–88 years	60/60	3–5 years	4, 14, 23F (Merck-ELISA; OPA)	Funded in part by Merck & Co and the CDC
Musher, 2010 [12]	USA	Resident population ≥ 50 year	437/544^5^	3–5 years	3, 4, 6B, 8, 9V, 12F, 14, 23F (Merck-ELISA)	Merck & Co
Musher, 2011^6^ [28]	USA	Resident population 60–93 years	67 (2nd dose)/67 (3rd dose)	10 years	3, 4, 6B, 8, 9V, 12F, 14, 23F (Merck-ELISA)	Merck & Co
Ohshima, 2014 [29]	Japan	Paitents with COPD, 65–80+ yrs	40 (longitudinal cohort)	8–9 years	6B, 14, 19F, 23F (WHO-ELISA; OPA)	Ministry of Health, Labour, and Welfare of Japan
Törling, 2003 [24]	Sweden	Patients with history of CAP, 50–88 years	61 (longitudinal cohort)	4–7 (mean 5.3) years	1, 4, 7F, 14, 18C, 19F (combined GMCs, non-WHO-ELISA)	not reported
Retrospective database studies (*n* = 3)						
Jackson, 2006 [27]	USA	Resident population, 50–80+ yrs	279,504/36,888 (2nd dose) and 603 (3rd dose)	1–9+ years (mean after 2nd dose 7 (±3) years)	Safety outcomes only	Not reported
Shih, 2002 [30]	USA	Resident population, 65–80+ yrs	96,327/23,663	6 months-9 years (43% > 5 years)	Safety outcomes only	Centers for Medicare & Medicaid Services
Walker, 2005 [32]	USA	Alaska native population, 72% ≥ 60 year	144/35 (2nd dose) and 179 (≥3rd dose)	45% ≥ 6 years, 55% < 6 years	Safety outcomes only	Funded in part by Association of Schools of Public Health
Cross-sectional study (*n* = 1; telephone interview)						
D’Heilly, 2002 [26]	USA	Elderly resident population, mean 71 year	455/107	not reported	Safety outcomes only	Not reported

*COPD* chronic obstructive pulmonary disease, *ELISA* enzyme linked immunosorbent assay, *WHO* World Health Organization, *OPA* Functional antibody activity assay, *CDC* centers for disease control and prevention, *CAP* community acquired pneumonia

^1^In some studies, some analyses (e.g. safety) were conducted in smaller subpopulation; ^2^Published as randomized controlled trial but treated as cohort study here; ^3^ Not all patients were considered for all endpoints; ^4^Substudy of Musher et al. [12]; ^5^ Number of participants at 5 years: 308/243; ^6^Extension study of Musher et al. [12]

In 6 studies two different study groups were compared: one group consisted of participants who had received a primary PPSV-23 dose, and the second group comprised those who had received one [12, 13, 23, 25] or more than one [26, 30] PPSV-23 revaccination doses after 3–13 years. One study compared participants who had received at least three PPSV-23 doses as compared to those who had received a primary or second dose of PPSV-23 [32]; two studies [11, 27] compared three study groups in which participants had received a first, second or third dose of PPSV-23 (after 1–22 years). In most included studies, participants of the revaccination group were older and/or had more underlying comorbidities as compared to participants who received a primary PPSV-23 dose (see Table 2).

**Table 2:** Characteristics of included studies (continued)

Author, year [Ref]	Number of study groups	Safety assessment	Statistically significant differences in baseline characteristics and safety outcomes between primary and revaccination dose of PPSV-23
Prospective cohort studies (*n* = 10)			
Tobudic, 2012^1^ [31]	1 (longitudinal cohort)	7 day diary (after revaccination dose)	Population characteristics: Participants 1 year older at 2^nd^ dose
			Safety: no comparison group
Dransfield, 2012^1^ [23]	2 (1^st^ vs. 2^nd^ dose)	not assessed	Population characteristics: 2^nd^ dose recipients older, more often white, more severe COPD disease
			Safety: −
Hammitt, 2011 [11]	3 (1^st^ vs. 2^nd^ or 3^rd^ dose)	4 day diary and interview on day 30	Population characteristics: 2^nd^/3^rd^ dose recipients older, more likely Alaska Natives/American Indians, more often with underlying comorbidities compared to 1^st^ dose recipients
			Safety: local AEs and systemic AEs more frequent in revaccination group
Jackson, 1999 [13]	2 (1^st^ vs. 2^nd^ dose)	13 day diary and telephone interview	Population characteristics: 2^nd^ dose recipients more often females and less often with underlying comorbidities
			Safety: local AEs more frequent in revaccination group at days 0–2, no differences after 6 days. No differences regarding systemic AEs. Multivariate analysis: revaccination independently associated with risk of sizable local reaction
Jackson, 2013 [22]	1 (longitudinal cohort)	13 day diary	Population characteristics: Participants 3.4–5 years older at 2^nd^ dose
			Safety: local AEs and systemic AEs more frequent in revaccination group
Manoff, 2010^2^ [25]	2 (1^st^ vs. 2^nd^ dose)	not assessed	Population characteristics: 2^nd^ dose recipients more likely ever smoked
			Safety: −
Musher, 2010^3^ [12]	2 (1^st^ vs. 2^nd^ dose)	14 day diary	Population characteristics: 2^nd^ dose recipients more often with underlying comorbidities
			Safety: local AEs and systemic AEs more frequent in revaccination group
Musher, 2011^3,4^ [28]	2 longitudinal cohorts (1^st^ vs. 2^nd^; 2^nd^ vs. 3^rd^)	14 day diary	Population characteristics: Participants of both longitudinal cohorts were ten years older at 2^nd^/3^rd^ dose
			Safety^5^: local AEs and systemic AEs more frequent in revaccination group
Ohshima, 2014 [29]	1 (longitudinal cohort)	14 day diary	Population characteristics: Participants were 7.6 years older at 2^nd^ dose
			Safety: local AEs and systemic AEs more frequent in revaccination group
Törling, 2003 [24]	1 (longitudinal cohort)	not assessed	Population characteristics: Participants were 5.3 years older at 2^nd^ dose and 11% had a new episode of pneumonia
			Safety: no comparison group
Retrospective database studies (*n* = 3)			
Jackson, 2006 [27]	3 (1^st^ vs. 2^nd^ vs. 3^rd^ dose)	ICD-9-Codes	Population characteristics: 3^rd^ dose recipients were older and had more likely underlying comorbidities
			Safety: Presumptive medically attended injection site reaction more frequent in 2^nd^ dose recipients than in 1^st^ dose recipients. No statistically significant differences between 1^st^ dose and 3^rd^ dose recipients
Shih, 2002 [30]	2 (1^st^ vs. ≥ 2^nd^ dose)	ICD-9-Codes	Population characteristics: 2^nd^ dose recipients were older, more often white and had higher hospitalizations rates and a higher comorbidity (Charlson) Index
			Safety: Mulitivariate analysis: Revaccination independently associated with emergency room visits and office visits if PPSV-23 was administered within 5 years. No association after >5 years.
Walker, 2005 [32]	2 (1^st^ or 2^nd^ vs. ≥ 3^rd^ dose)	ICD-9-Codes and medical records	Population characteristics: ≥ 3^rd^ dose recipients were older and had more likely underlying lung diseases
			Safety: No differences in risk of medically attended AEs in the different groups
Cross-sectional study (*n* = 1; telephone interview)			
D’Heilly, 2002 [26]	2 (1^st^ vs. ≥ 2^nd^ dose)	Interview 8 months (on average) after vaccination	Population characteristics: not reported
			Safety: Multivariate analysis: Revaccination independently associated with redness or swelling at injection site during week after vaccination

^1^Published as randomized controlled trial but treated as cohort study here; ^2^Substudy of Musher et al. [12]; ^3^Musher [28] is extension study of Musher et al. [12]; ^4^ two longitudinal cohorts: cohort one received 1st dose in 1997 and 2nd in 2007; cohort two received 2nd dose in 1997 and 3rd in 2007; ^5^2nd vs. 3rd dose (1st vs. 2nd dose reported in Musher [12])

### Reported outcomes

None of the studies reported effectiveness of PPSV-23 against clinical outcomes. Three studies [23–25] reported immunogenicity data, four studies [26, 27, 30, 32] reported safety data and seven studies [11–13, 22, 28, 29, 31, 33] reported both. Three publications reported data from partially overlapping study populations [12, 25, 28]; however, data from non-overlapping subgroups were extracted from all three papers. The time interval from primary to revaccination varied widely within and between studies with a range from 6 months to 22 years (Table 1). Although in the study of Shih et al. [30] some participants received the revaccination dose within one year, this study was not excluded, since this subgroup of vaccinees represented less than 10% of the entire study population.

### Risk of bias in individual studies

In 11 (78%) studies risk of bias was high [11–13, 22–24, 26, 28, 29, 31, 32]. This was mainly due to insufficient control for confounders or due to a high risk of selection bias during the recruitment process. In the remaining three studies [25, 27, 30], risk of bias was unclear owing to non-specific disease diagnosis codes (ICD-9 codes) collected within a passive surveillance system or since selection bias might have occurred during recruitment of the study participants.

### Immunogenicity of primary vaccination and revaccination with PPSV-23

Nine studies used enzyme-linked immunosorbent assays (ELISAs) to measure serotype-specific anti-pneumococcal immunoglobulin G (IgG) levels, but only three studies [23, 29, 31] followed the current World Health Organization (WHO)-approved ELISA protocol (Table 1). Four studies [11, 23, 25, 29] additionally assessed functional antibody activity assay (OPA). One study [22] reported OPA results only. The number of analyzed serotypes ranged from 3 to 13. One study [24] reported only combined GMCs against six different serotypes and two studies did not provide data on 95% CIs [23, 25].

**Fig. 2:**
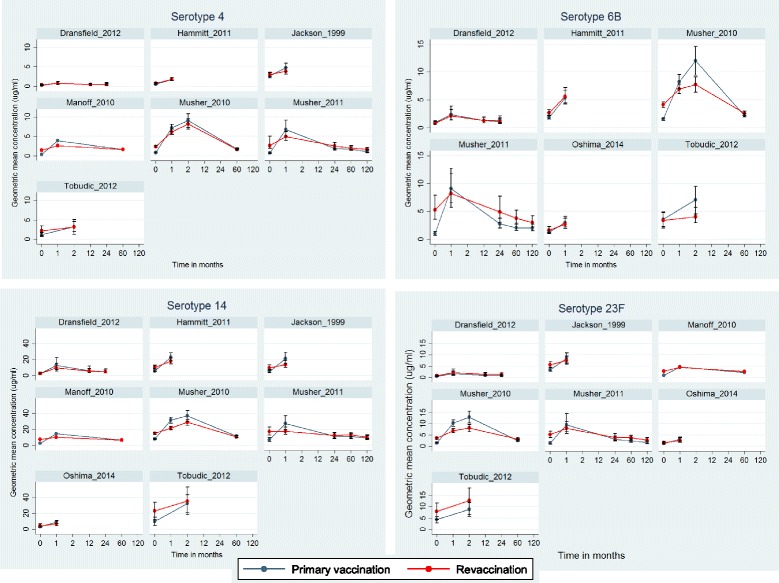
Geometric mean concentrations (GMC) with 95% confidence intervals of the most commonly analyzed serotypes (4, 6B, 14, 23F)

Within studies, serotype-specific GMCs differed widely between serotypes (see Fig. 2 Generally, GMCs tended to be higher for serotype 14 compared to other serotypes. Differences in serotype-specific GMCs were also obvious between the different studies. For example, GMCs measured by Musher et al. [12] were generally higher than GMCs measured in the studies of Dransfield et al. [23], Ohshima et al. [29], or Hammitt et al. [11]. In all studies, GMCs tended to decline rapidly after an initial peak at 1 to 2 months after both primary vaccination and revaccination.

**Fig. 3:**
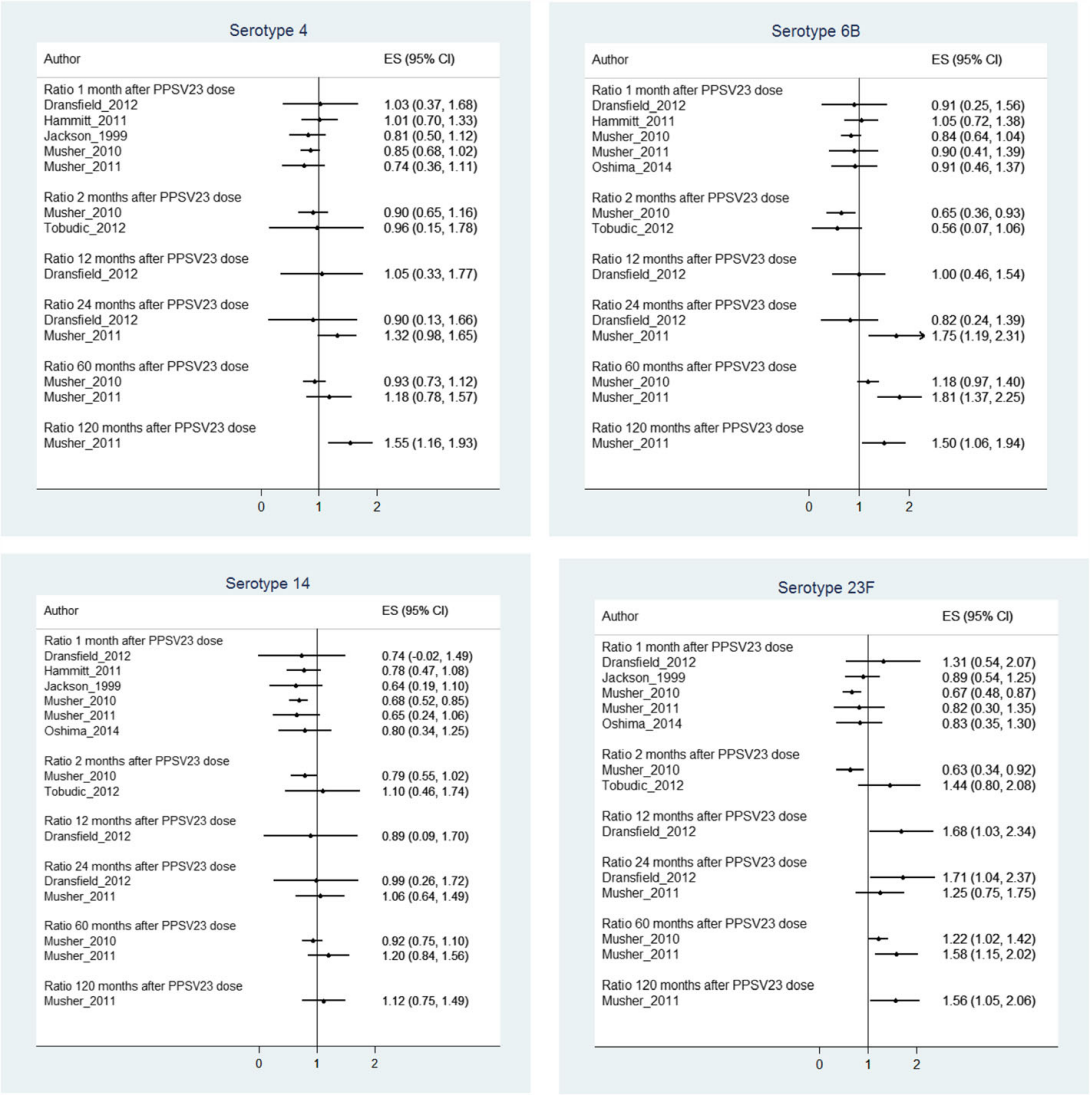
Ratios (2^nd^ dose/1^st^ dose) of geometric mean concentrations (GMC) with 95% confidence intervals of the most commonly analyzed serotypes (4, 6B, 14, 23F)

One to two months after (re-)vaccination, GMC ratios (GMC after second dose divided by GMC after first dose) were mostly below 1, indicating higher short-term immunogenicity of the first PPSV23 dose as compared to the second dose, although these results were statistically not significant (Fig. 3 and Additional file 1). At later time points, GMC ratios for most serotypes were not different from 1, indicating that the ensuing long-term immune responses to primary vaccination and revaccination did not differ. In four studies in which PPSV-23 antibodies were investigated over a period of two or more years [12, 23, 25, 28], GMC ratios for some serotypes (serotype 6B and 23F) increased to >1 (Fig. 2). In the studies of Musher et al., GMC ratios increased to >1 in the majority of analyzed serotypes 5 to 10 years after vaccination [12, 28]. Although absolute antibody levels decreased continually over time, they remained higher than the pre-vaccination levels before primary vaccination or revaccination. Ratios could not be calculated for three studies since either absolute numbers or 95% CIs were not available [23–25].

OPA data were available for analysis from 4 of 5 studies and revealed contradictory results. In the study of Hammitt et al. [11], 2 of 5 serotypes showed greater immune response one month after revaccination dose than after the primary dose given 6–22 years earlier (see Additional file 1). Jackson et al. [22] found that OPA immune response after primary vaccination was significantly higher in 9 of 14 analyzed serotypes compared to revaccination 3–4 years later. Manoff et al. found for the three investigated serotypes comparable OPA values one month after primary and secondary vaccination [25]. Finally, in the study of Ohshima et al., no statistically significant differences of OPA values were found in 4 analyzed serotypes [29]. One study did not provide data of a primary PPSV-23 dose as compared to revaccination [23].

Taken together, these results suggest that revaccination with PPSV-23 may induce a less pronounced rise of antibody levels than the primary vaccination in the first 2 months after vaccination, which, however, does not impair long-term persistence of anti-polysaccharide antibodies.

### Safety of primary vaccination and revaccination with PPSV-23

Ten of 14 studies compared frequency and/or severity of AEs after primary and revaccination doses with PPSV-23 (see Table 1). In four studies, safety of ≥ 3 doses was assessed [11, 27, 28, 32]. Safety data were collected through patient diaries [11–13, 22, 28, 29], by analyzing ICD-9 codes and/or medical records of vaccinated subjects [27, 30, 32] or by telephone interview of vaccinees [26].

There was a wide range of reported safety endpoints ranging from two [31] to seven [13] local AEs, such as redness, swelling or limitation of arm movement, and from five [11, 28] to twelve [22] systemic AEs, such as arthralgia, fatigue, fever, headache, nausea, myalgia or rash. Additionally, local and/or systemic AEs were graded as mild, moderate or severe in the majority of the studies.

Frequency of local and systemic AEs differed widely between the six studies in which patient diaries were used [11–13, 22, 28, 29]. For example, fever ranged from 0 to 9% [22, 28] after the primary dose and from 2 to 10% [13, 22] after revaccination dose, any headache from 2 to 61% [22, 29] in primary vaccinated and from 13 to 57% [13, 22] in revaccinated individuals. Limitation of arm movement ranged from 4 to 31% [11, 22] after primary and from 13 to 47% [11, 22] after revaccination.

Of four studies in which occurrence of AEs in correlation with (higher) pre-vaccination antibody levels was assessed, three studies found an association [11–13] and one study did not [24]. Regarding the time span since previous vaccination, two [28, 30] of 5 studies found that a longer time period (>5 to 10 years) since primary vaccination reduced severity and/or frequency of AEs. The remaining studies [13, 26, 27] did not find a statistically significant association between time span since primary vaccination and severity of AEs.

In studies that used ICD-9 codes to assess differences in vaccine-related unplanned medical visits, differences were smaller and ranged from 0.3 to 1.3% after primary and from 0.7 to 1.9% after revaccination doses [27, 30, 32, 34].

Of four studies in which safety of a third dose was analyzed, one study found more local and systemic AEs among third-dose recipients than among second-dose recipients [28], whereas another study found no differences between second-dose and third- *or* fourth-dose recipients [11]. In the remaining two studies, no differences in medically attended AEs were identified after a third dose compared to a first *or* second dose [27, 32].

Four studies provided adjusted safety analyses [13, 26, 27, 30]. Jackson et al. found that revaccination with PPSV-23 was independently associated with the risk of a sizable local reaction [13]. In this study, the majority of local adverse events disappeared within 6 days of vaccination. Shih et al. identified revaccination with PPSV-23 as a risk factor for emergency department visits or medical office visits [30]. In the Vaccine Safety Datalink population in the U.S., elderly with a second PPSV-23 dose had a higher risk of a *presumptive medically attended injection site reaction* than those who received a primary *or* a third dose [27]. D’Heilly et al. found that revaccination was independently associated with redness or swelling at the injection site [26].

Overall, local AEs and systemic AEs after revaccination were reported more frequently in studies in which patient diaries were used (Table 1). Two of four studies found that unplanned medical visits were more frequent after a revaccination dose [27, 30]. None of the studies reported serious adverse events during the observation periods after primary vaccination or revaccination, respectively.

## Discussion

Comparative data on the effectiveness of primary vs. revaccination with PPSV-23 are still lacking. Since we did not identify studies with clinical endpoints, evidence had to be derived from immunogenicity studies only. These studies indicated that antibody responses are reduced during the first months after revaccination as compared to primary vaccination with PPSV-23. However, differences were observed only for some serotypes and were restricted to early time points (i.e. one or two months after revaccination). Thereafter immunogenicity of primary vaccination and revaccination was comparable. After a period of more than two years antibody levels against some serotypes were even higher after revaccination than after primary vaccination. Regarding safety, our systematic review indicates that revaccination with PPSV-23 is associated with a higher frequency of local and systemic, self-limiting adverse events, but not with severe sequelae.

Measuring the efficacy/effectiveness against clinical outcomes has been advocated as the best way to assess the protective effect of immunizations [35, 36]. However, in the absence of such data, evidence has to be derived from immunogenicity studies that evaluate antibody responses after immunization. For PCV, a working group of the World Health Organization has defined in 2005 a mean concentration of ≥0.35 μg/ml of serotype-specific anti-polysaccharide IgG measured with ELISA one month after immunization as a “correlate of protection” against IPD in children [37]. However, it is unclear (i) how well this threshold correlates with the efficacy of a pneumococcal vaccine against clinical outcomes [36], (ii) whether this threshold also applies to polysaccharide vaccines and (iii) whether this threshold is also appropriate for the adult or the elderly population. In addition, since Andrews et al*.* showed that serotype-specific protection varies widely [38], serotype-specific thresholds might be necessary. On the basis of the proven protective efficacy of a primary vaccination with PPSV-23 [39, 40], we believe that demonstration of comparable immunogenicity more than 2 months after primary or revaccination indicates that protective efficacy should also be comparable and that an exact threshold is not essential.

In addition to serotype-specific IgG, measurement of functional antibodies as assessed by OPA is considered as a valid surrogate parameter for vaccine protection from pneumonia or bacteremia [23, 41, 42]. We identified 5 studies which assessed functional antibody activity and 4 of those provided enough data to calculate OPA ratios. According to these ratios, no uniform pattern was observed. Although Jackson et al. demonstrated that OPA immune responses one month after revaccination were lower than responses after primary vaccination [22], such a pattern was not reported by the other studies. In fact, Hammitt et al. found OPA immune responses to be even higher one month after revaccination in 2 of 5 serotypes [11]. Whether these inconsistencies result from differences in the study population or laboratory methods, could not be clarified with the available data.

It remains unclear why the rise in antibody levels after revaccination with pneumococcal polysaccharide vaccine is lower than after primary vaccination and if this might affect clinical effectiveness [10]. Several mechanisms have been discussed. For example, large amounts of polysaccharides might deplete memory B cells and B1b cells [43, 44], although this was not seen in other studies [45, 46]. Alternatively, other immune cells such as dendritic cells [47] or T lymphocytes with suppressor activity [48–50] might reduce immunological response to polysaccharides. Given these uncertainties, studies measuring clinical endpoints after revaccination with PPSV-23 are urgently needed.

Data on the safety of a revaccination vs. primary PPSV-23 dose were more consistent and indicate that revaccination with PPSV-23 is associated with a higher number of non-severe AEs as compared to the primary dose. However, since in the majority of studies participants of the revaccination groups were older and/or had more comorbidities than the comparison group, interpretation of the unadjusted rates of AEs has to be taken with caution. The fact that revaccination increases the risk of AEs is consistent with Arthus-type hypersensitivity reactions in which antigen-antibody complexes cause local symptoms after (re-)vaccination [13, 51]. Since this phenomenon requires residual antibodies, it seems plausible that a longer time span since the previous vaccination - accompanied by declining antibody levels - will reduce these reactions. This has been shown in 2 of 5 studies that investigated a time-depended frequency or severity of AEs and in 3 of 4 studies in which a correlation between pre-vaccination GMCs and (local) AEs was found. Therefore, revaccination after a longer time period (e.g. > 5 years) seems appropriate.

### Strengths and weaknesses of this study

This study has several strengths. It is based on a comprehensive systematic review of the currently available literature, comprising immunogenicity as well as safety data. By calculating the ratios of GMTs, we provide a measure of relative immunogenicity of revaccination, which is independent of differences of laboratory assays used in different studies. The usefulness of this approach was limited by differences in the choice and numbers of serotypes analyzed in different studies.

Interpretation of the immunogenicity data was complicated by differences in the composition of study populations (e.g., different age-groups, patients with COPD vs. resident population vs. renal transplant recipients) and differences regarding the follow-up period and time points at which blood sampling was performed. In addition, risk of bias was high in the majority of studies, limiting the internal validity of the results. Since no study reported adjusted immunological data, it remains unclear to what extend confounders such as comorbidities might have influenced the results. Furthermore, in some studies it remained unclear how selection of participants into the cohorts was performed. For these and further reasons related to heterogeneity of the study designs, it was not possible to calculate meta-analytical estimates.

## Conclusion

In conclusion, comparative data on the effectiveness of primary vs. revaccination with PPSV-23 are still missing, mainly due to the lack of randomized controlled trials using clinical endpoints. The available evidence from observational studies indicates that revaccination with PPSV-23 is likely to induce long-term antibody levels that are comparable to those after primary vaccination. However, due to differences in study design, study populations, different intervals between doses and high or unclear risk of bias in the included studies, these results have to be taken with caution and it remains unclear how immunogenicity results translate into clinical protection. Since primary vaccination with PPSV-23 has been shown to protect from pneumococcal disease, it seems likely that – based on similar antibody levels - also revaccination may confer. In view of the waning immunity and increasing risk of severe pneumococcal disease with age, the benefits of revaccination with PPSV-23 probably outweigh the increased risk of non-severe local and systemic AE. Revaccination could therefore be considered in the elderly after informing about benefits and possible harms of revaccination. In order to obtain strong evidence for the effectiveness of PPSV-23 in the elderly, RCTs or carefully designed observational studies using clinical relevant endpoints are urgently needed.

## Additional file

Additional file 1: Search strategy for the manuscript entitled “Effectiveness, immunogenicity and safety of 23-valent pneumococcal polysaccharide vaccine revaccinations in the elderly: a systematic review”. List of excluded studies (full-texts) on effectiveness, immunogenicity and safety of 23-valent pneumococcal polysaccharide vaccine revaccinations in the elderly. Ratios (2^nd^ dose PPSV23/1^st^ dose PPSV23) of geometric mean concentrations (GMC) of pneumococcal serotypes. Ratios (2^nd^ dose PPSV23/1^st^ dose PPSV23) of opsonophagocytic assays (OPA). (DOCX 816 kb)

## Abbreviations

AE: Adverse events
GMC: Geometric mean concentrations
NITAG: National Immunization Technical Advisory Group
OPA: Functional antibody activity assay
OPI: Opsonophagocytic index
PCV-13: 13-valent pneumococcal conjugate vaccine
PPSV-23: 23-valent pneumococcal polysaccharide vaccine
PRISMA: Preferred reporting items for systematic reviews and meta-analysis
RCT: Randomized controlled trial
