# Supplementary material for: Effectiveness, immunogenicity and safety of 23-valent pneumococcal polysaccharide vaccine revaccinations in the elderly: a systematic review
Source: BMC Infect Dis. 2016 Nov 25;16:711. doi: 10.1186/s12879-016-2040-y (PMC5124290; doi:10.1186/s12879-016-2040-y)
Supplement: Additional file 1: — Search strategy for the manuscript entitled “Effectiveness, immunogenicity and safety of 23-valent pneumococcal polysaccharide vaccine revaccinations in the elderly: a systematic review”. List of excluded studies (full-texts) on effectiveness, immunogenicity and safety of 23-valent pneumococcal polysaccharide vaccine revaccinations in the elderly. Ratios (2nd dose PPSV23/1st dose PPSV23) of geometric mean concentrations (GMC) of pneumococcal serotypes. Ratios (2nd dose PPSV23/1st dose PPSV23) of opsonophagocytic assays (OPA). (DOCX 816 kb) [file 12879_2016_2040_MOESM1_ESM.docx]

**Appendix (additional files 1-4)** to the manuscript entitled “Effectiveness, immunogenicity and safety of 23-valent pneumococcal polysaccharide vaccine revaccinations in the elderly: a systematic review” by Cornelius Remschmidt et al.

**Additional file 1**: Search strategy for the manuscript entitled “Effectiveness, immunogenicity and safety of 23-valent pneumococcal polysaccharide vaccine revaccinations in the elderly: a systematic review”

(Databases searched via the German Institute of Medical Documentation and Information (DIMDI, <https://www.dimdi.de/static/en/index.html> ): MEDLINE, EMBASE and Cochrane Central Register of Controlled Trials from inception to May 26, 2015; restrictions: species: human)

| **#** | **Search terms** | **Hits** |
| --- | --- | --- |
| #9 | check duplicates, unique in #8 | 1162 |
| #8 | #7, species human | 1891 |
| #7 | #1 AND #2 AND #3 AND #6 | 2051 |
| #6 | #4 OR #5 | 5357545 |
| #5 | (FT=antibod* OR (CT D "event, adverse drug" OR UT="event, adverse drug" OR IT="event, adverse drug" OR SH="event, adverse drug")) OR (CT D "efficiency" OR UT="efficiency" OR IT="efficiency" OR SH="efficiency") | 2286275 |
| #4 | (((FT=safety OR FT=reactogeni* ) OR FT=immunogenici* ) OR FT=effectiveness ) OR FT=efficacy | 3275243 |
| #3 | (((FT=vaccin* OR FT=immuni* ) OR FT=PSV ) OR (CT D "polysaccharide vaccine, pneumococcal" OR UT="polysaccharide vaccine, pneumococcal" OR IT="polysaccharide vaccine, pneumococcal" OR SH="polysaccharide vaccine, pneumococcal")) OR (CT D "immunization" OR UT="immunization" OR IT="immunization" OR SH="immunization") | 1238863 |
| #2 | (((FT=booster OR FT=revacc* ) OR FT=second ) OR FT=third ) OR (CT D "revaccination" OR UT="revaccination" OR IT="revaccination" OR SH="revaccination") | 2304430 |
| #1 | ((FT=pneumococc* OR FT=streptococcus pneumoniae ) OR (CT D "pneumococcus" OR UT="pneumococcus" OR IT="pneumococcus" OR SH="pneumococcus")) OR (CT D "streptococcus pneumoniae" OR UT="streptococcus pneumoniae" OR IT="streptococcus pneumoniae" OR SH="streptococcus pneumoniae") | 97630 |

Abbreviations: CT= controlled terms; FT= free text; IT=Index term; SH= section heading; UT=uncontrolled terms

**Additional file 2**: List of excluded studies (full-texts) on effectiveness, immunogenicity and safety of 23-valent pneumococcal polysaccharide vaccine revaccinations in the elderly (n= 28).

No serial vaccination with PPSV23 (e.g. PPSV23-PCV; n=12): [1-12]

Population < 50 yrs (n=5): [13-17]

No data on primary PPSV23 dose (n=4): [18-21]

Other vaccine than PPSV23 (n=2): [22, 23]

Study not found/ study with implausible data (n=2): [24, 25]

Study population already included in another study (Musher et al.; n=1): [26]

Duplicate (n=2)

**Additional file 3**: Ratios (2^nd^ dose PPSV23/ 1^st^ dose PPSV23) of geometric mean concentrations (GMC) of pneumococcal serotypes.

**3.1. Dransfield et al., 2012**


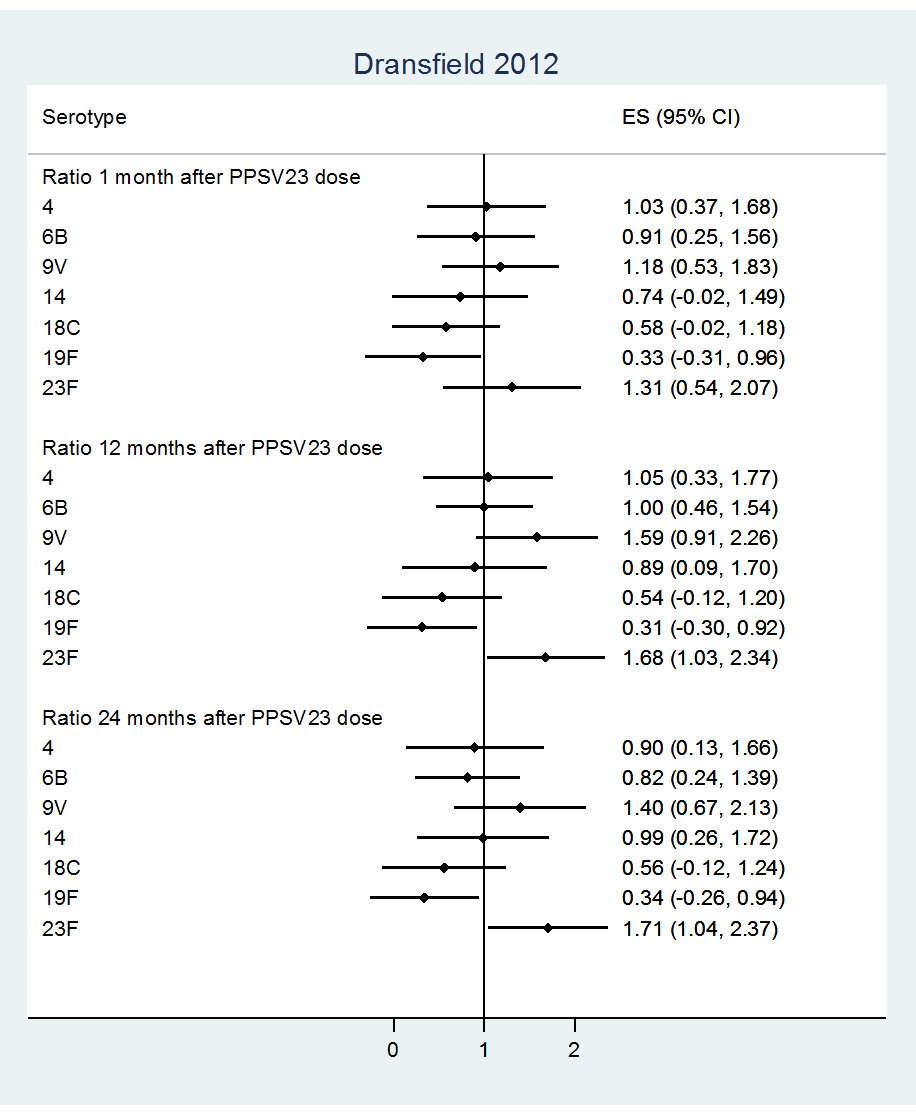


**3.2. Hammit et al., 2011.**


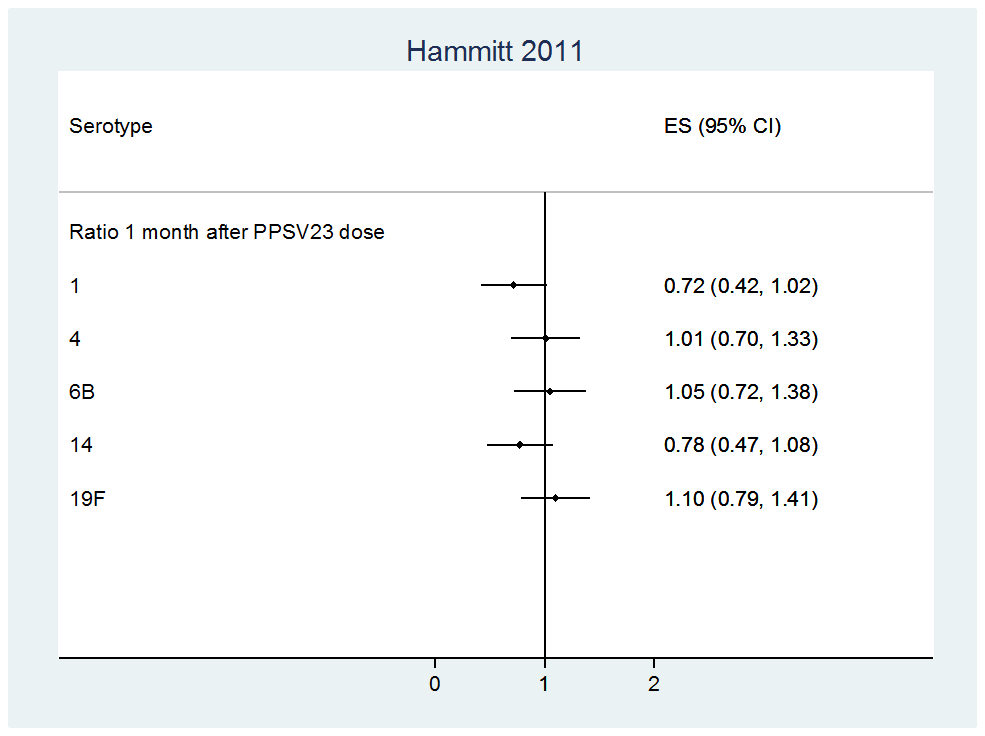


**3.3. Jackson et al., 1999.**

**3.4. Musher et al., 2010.**


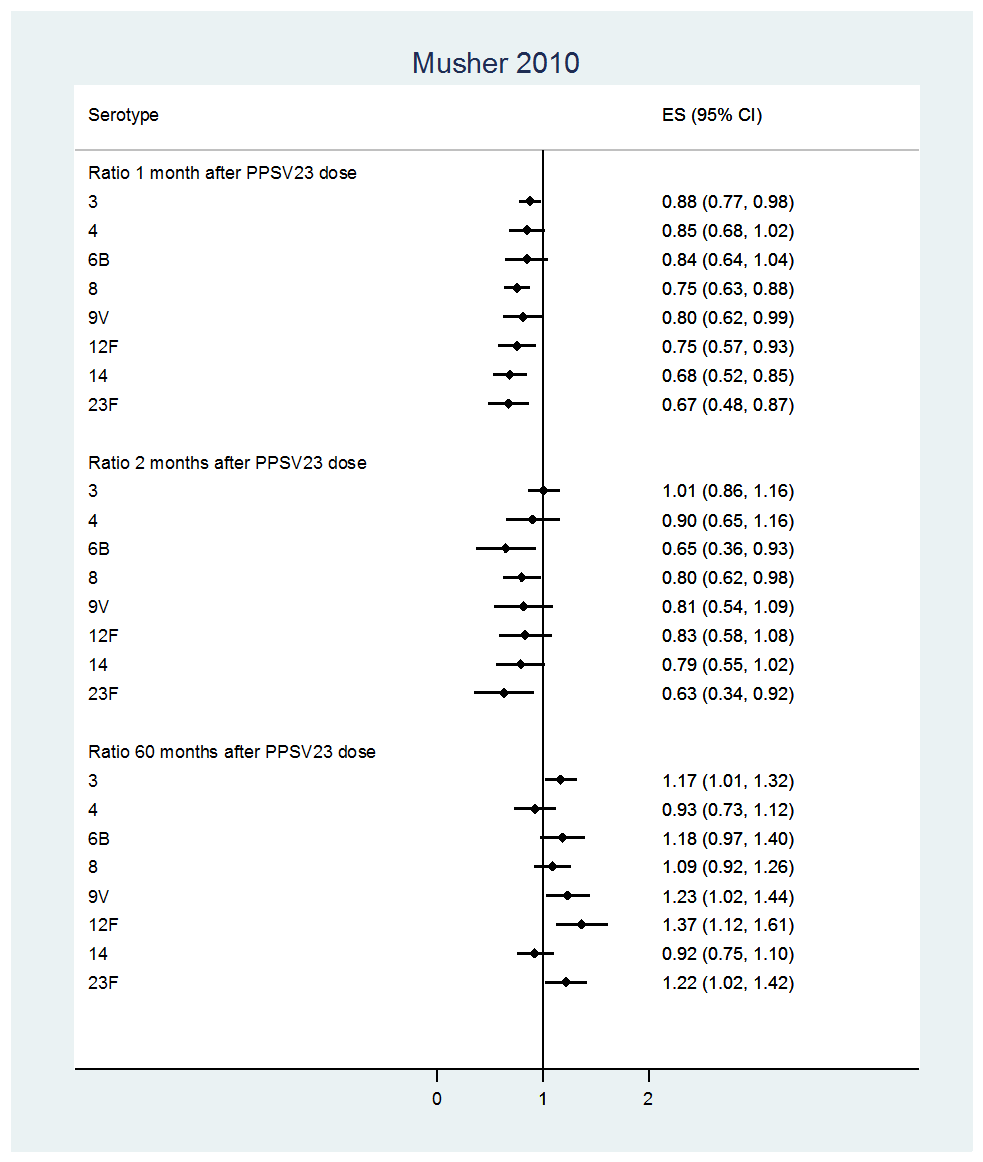
**3.5. Musher et al., 2011.**


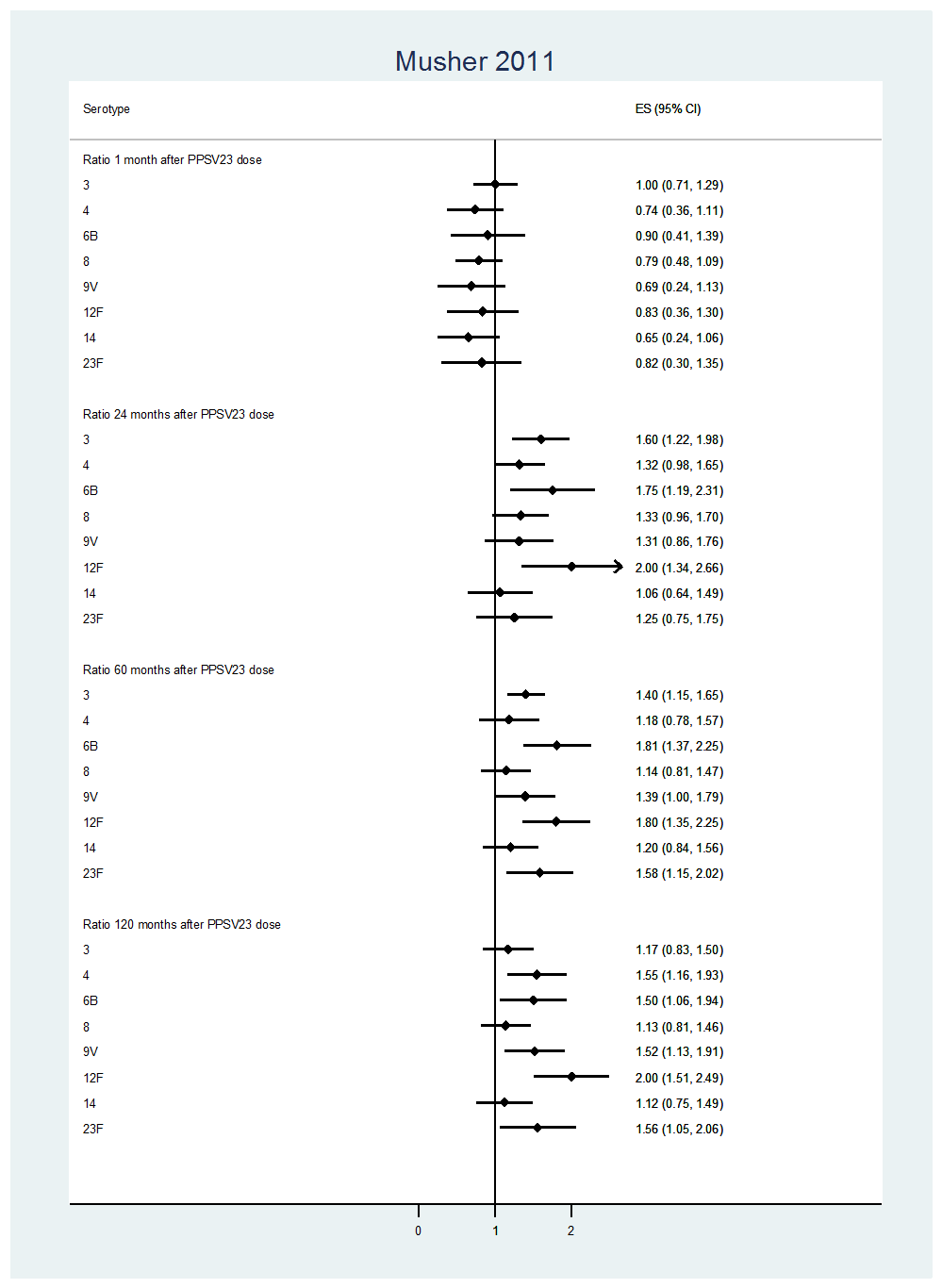


**3.6. Oshima et al., 2014**


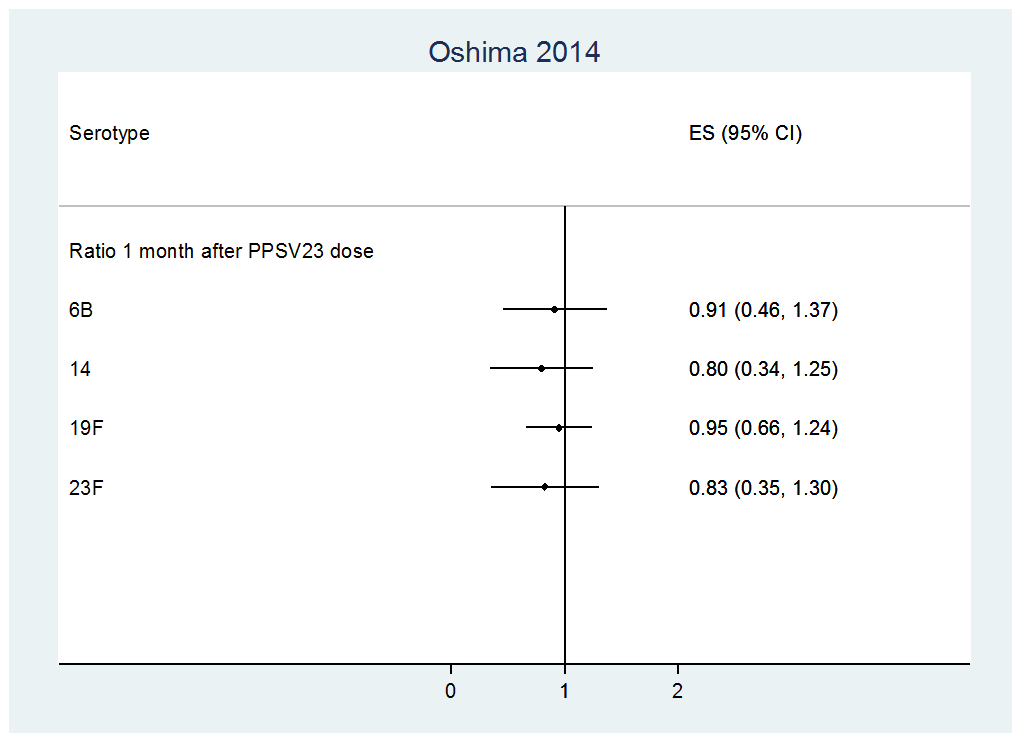


**3.7. Tobudic et al., 2012.**


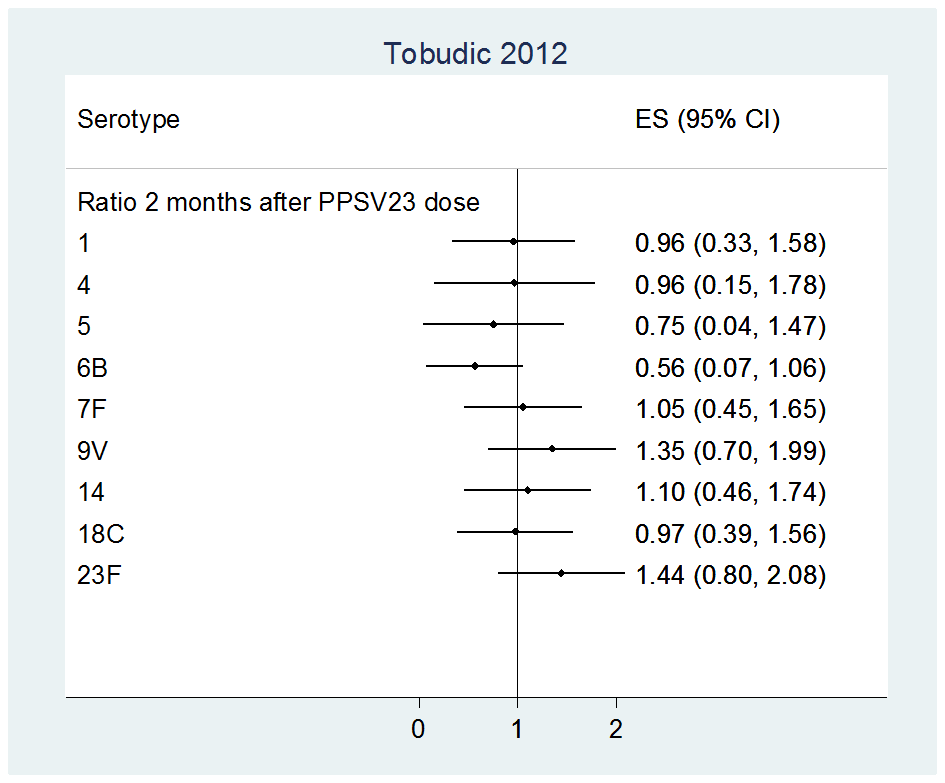


**Additional file 3a**: Ratios (2^nd^ dose PPSV23/ 1^st^ dose PPSV23) of geometric mean concentrations (GMC) of pneumococcal serotypes.

**3a.1 Serotype 1.**


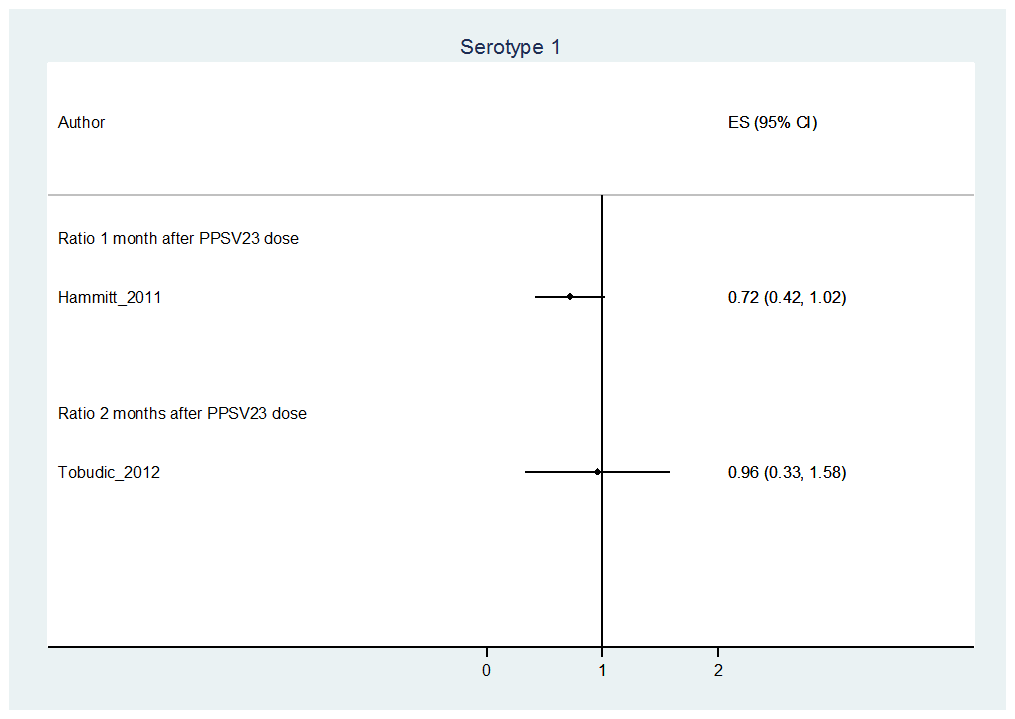


**3a.2. Serotype 3.**

**
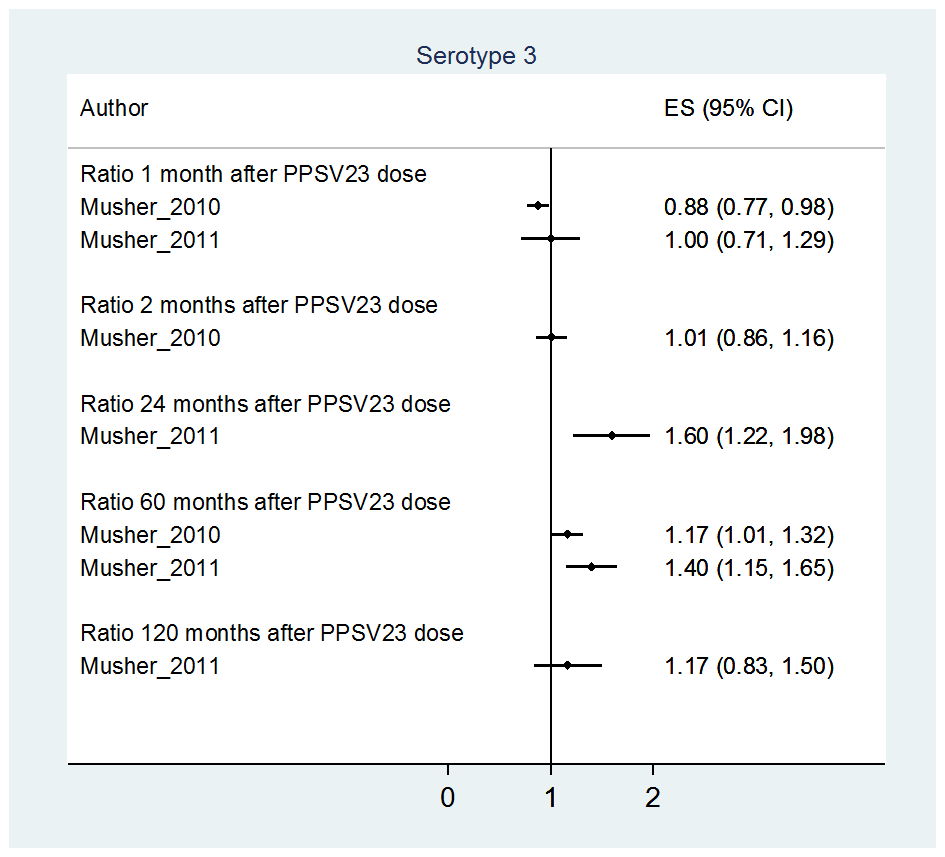
**

**3a.3. Serotype 4.**

**
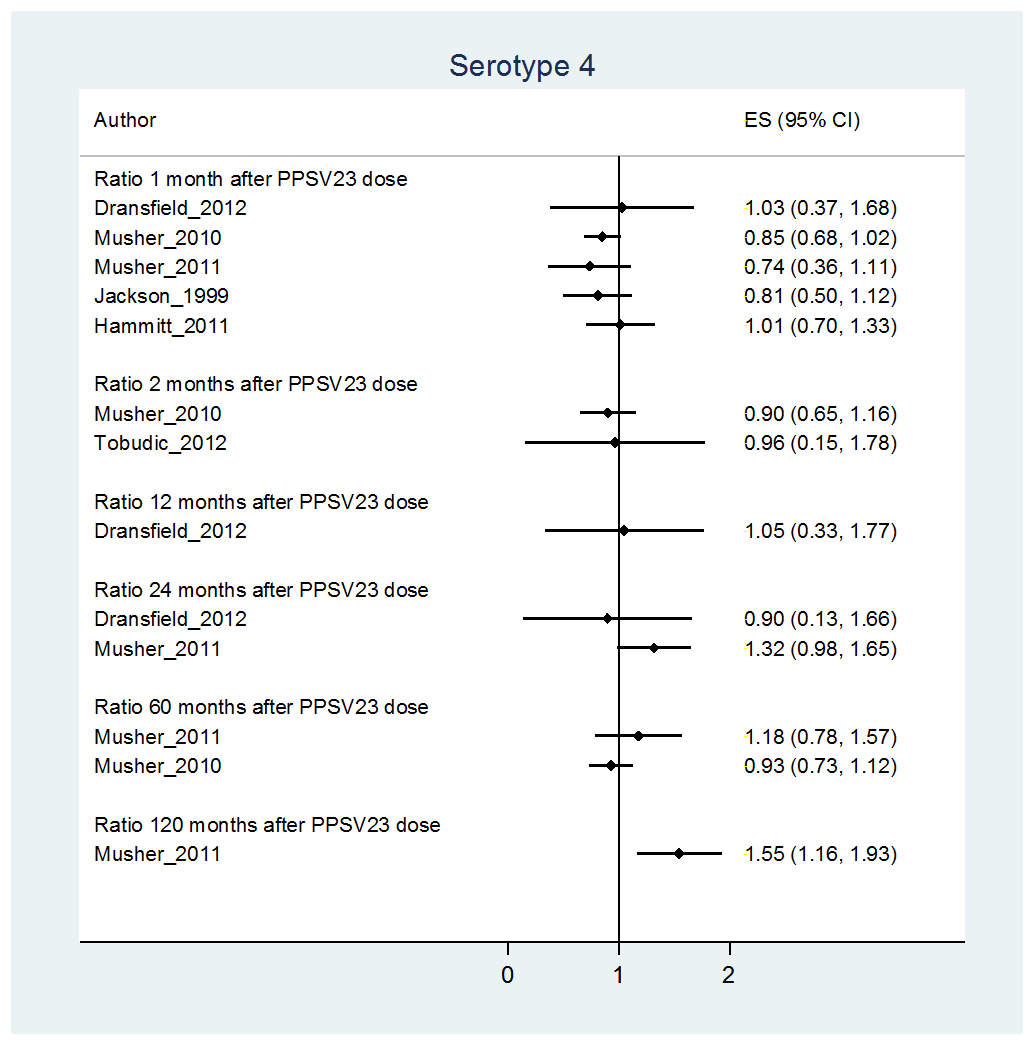
**

**3a.4. Serotype 6B.**


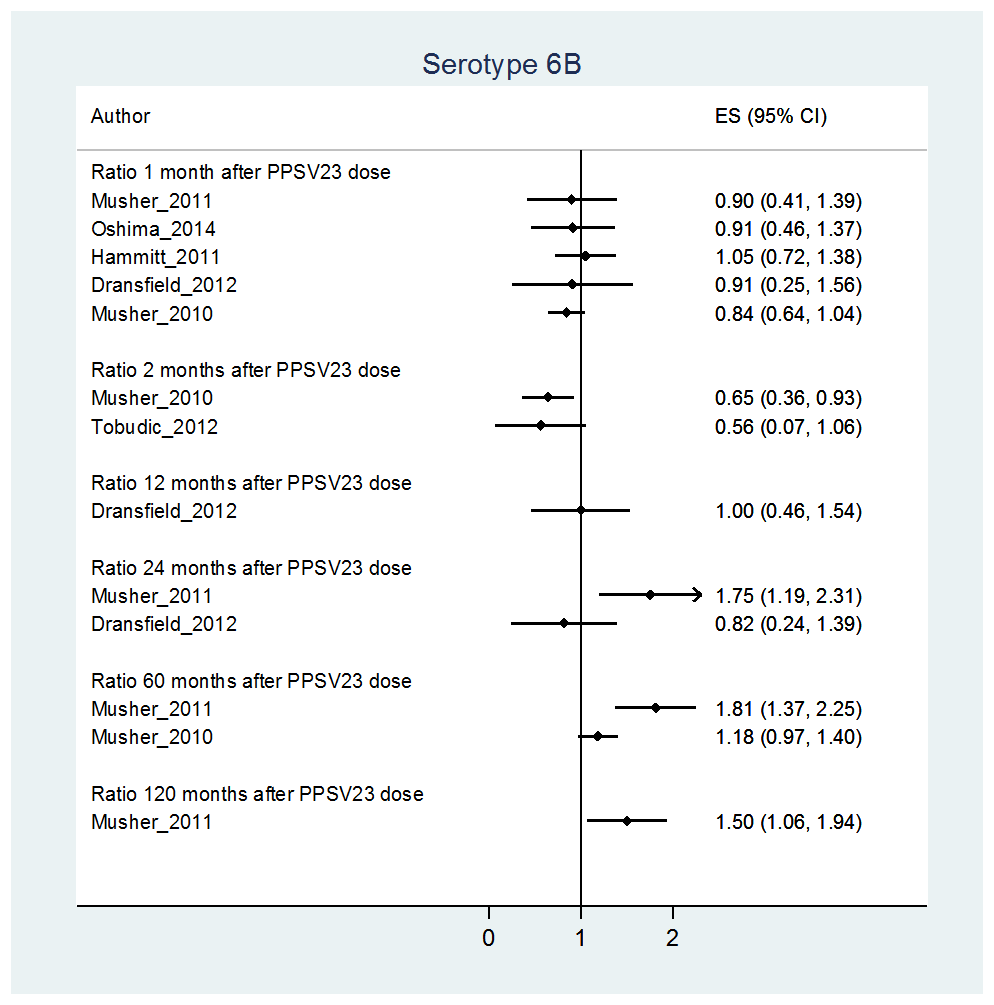


**3a.5. Serotype 8.**


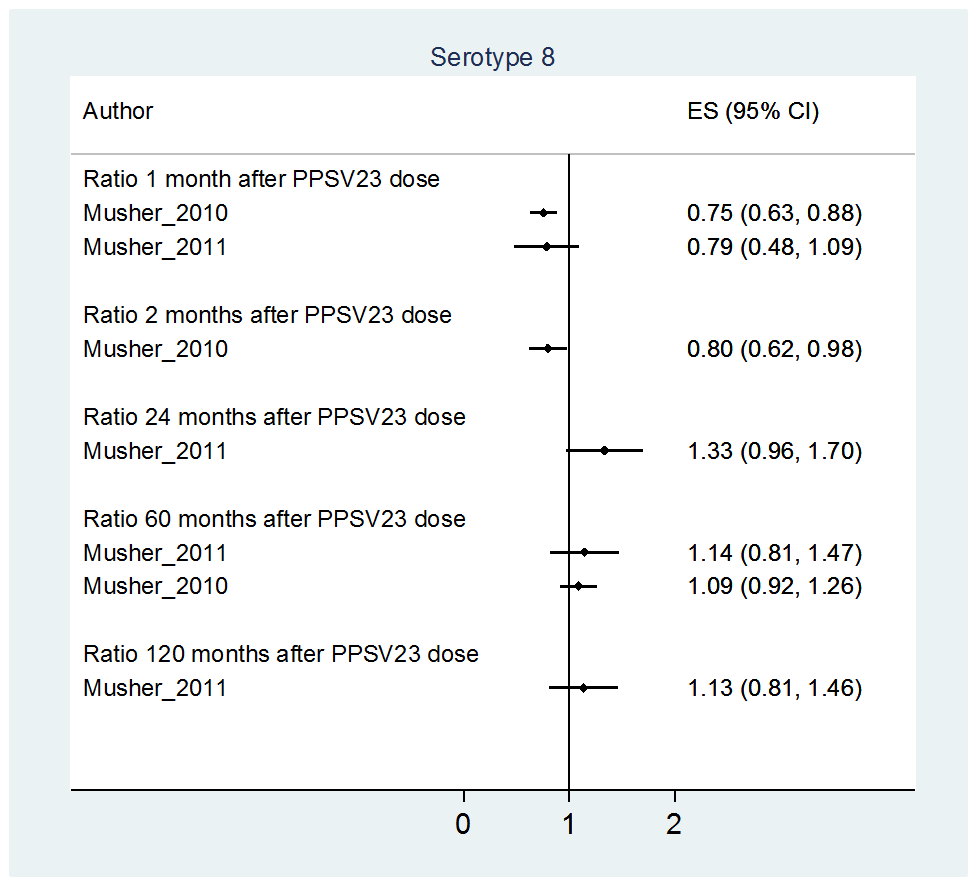


**3a.6. Serotype 9V.**


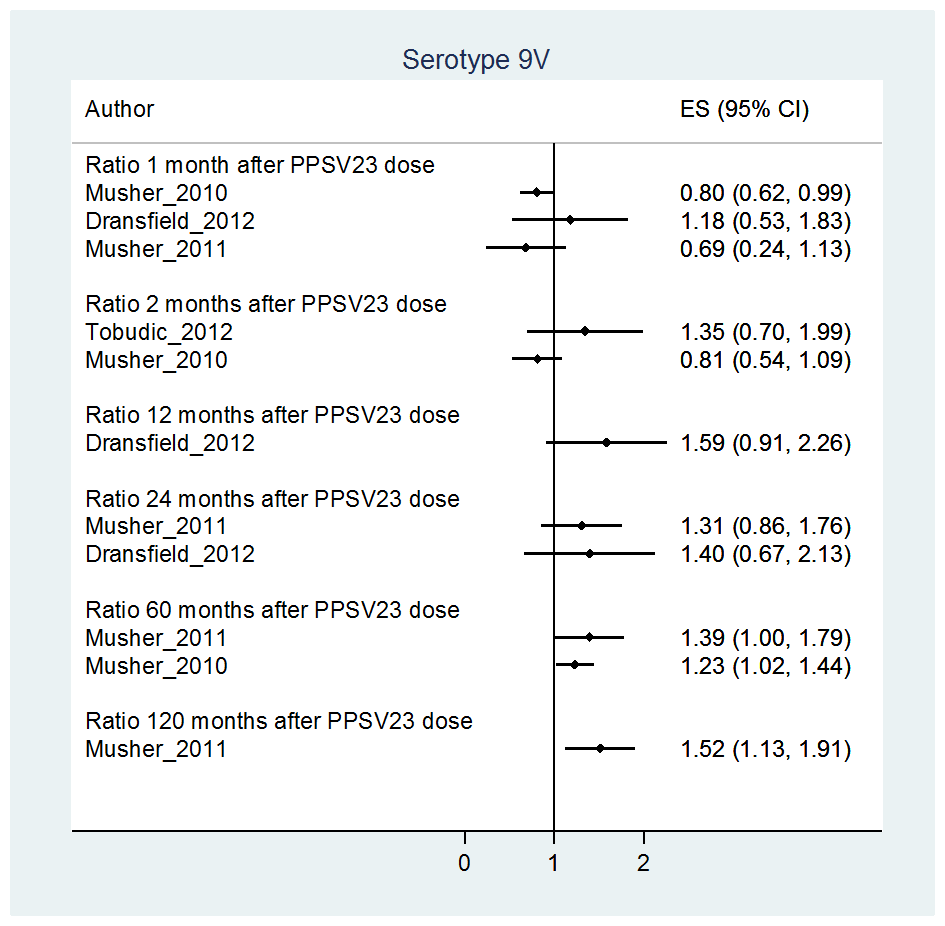


**3a.7. Serotype 12F.**


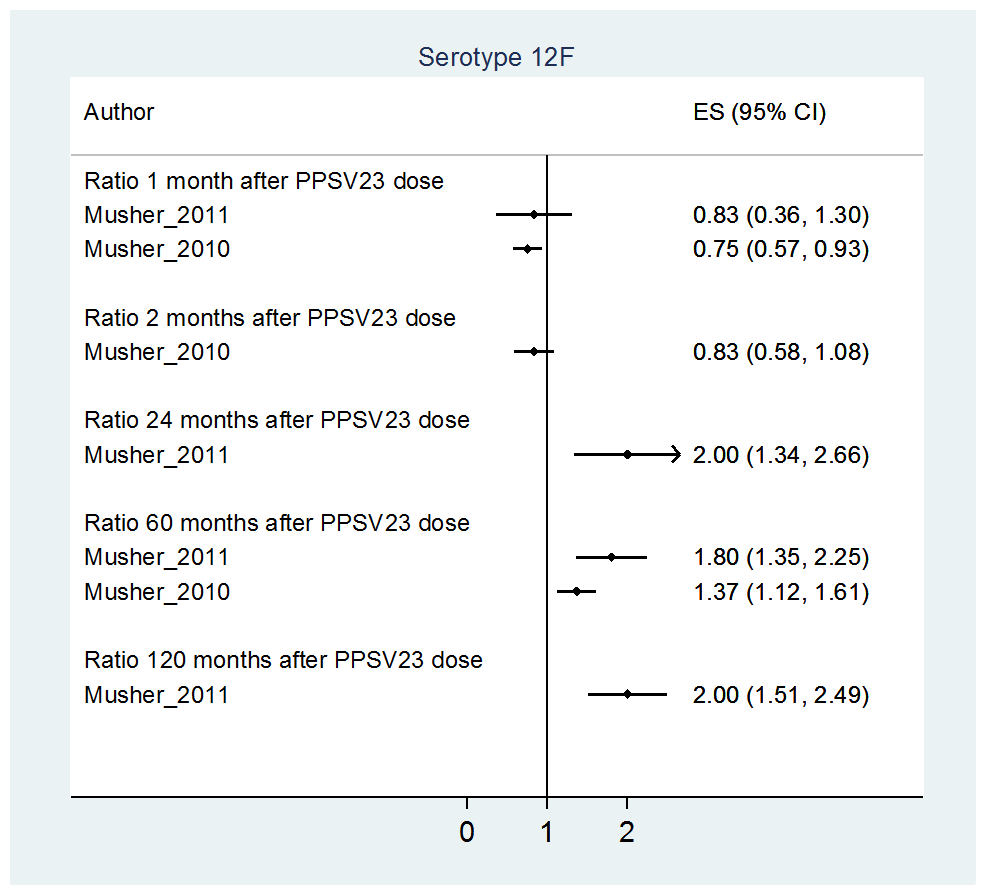


**3a.8. Serotype 14.**


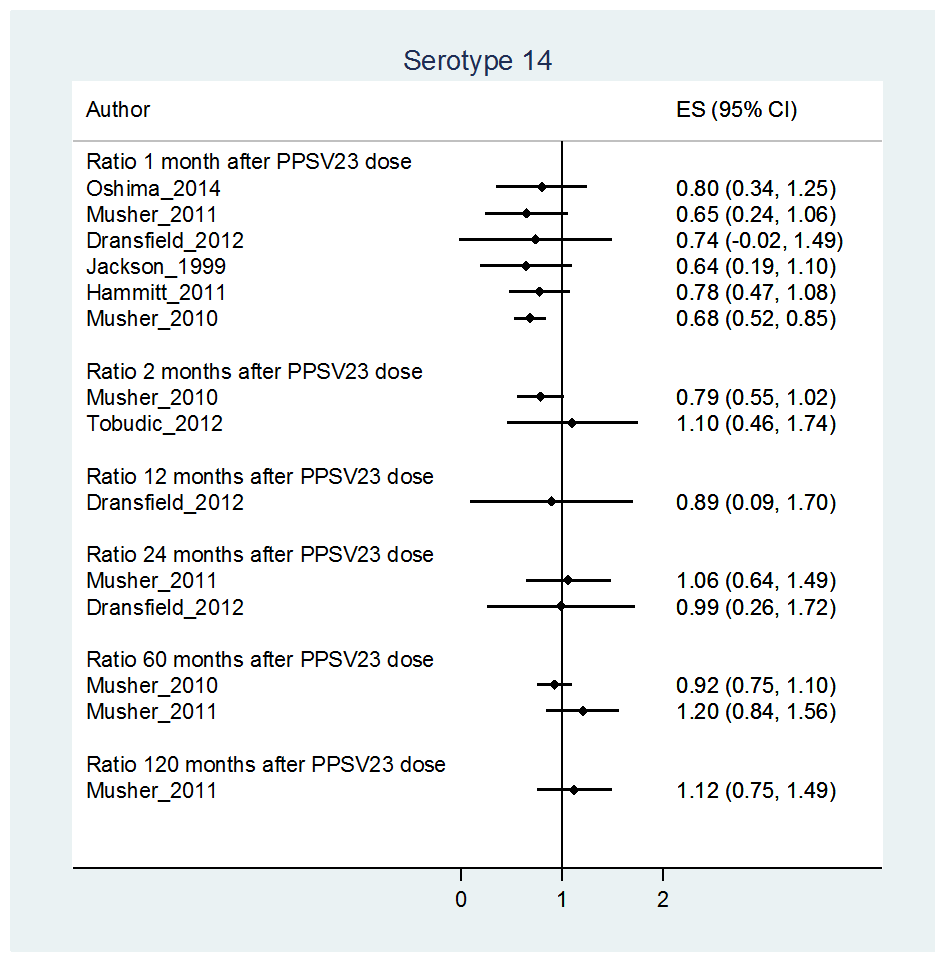


**3a.9. Serotype 18C.**

**3a.10. Serotype 19F.**


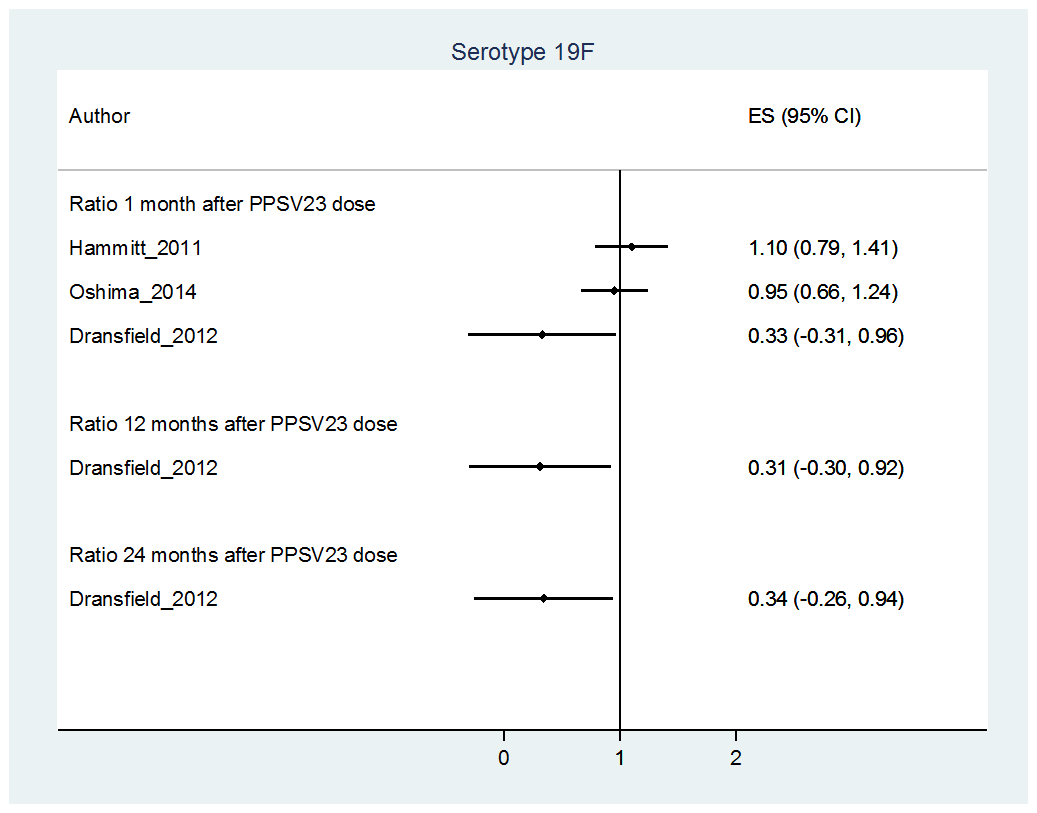


**3a.11. Serotype 23F.**


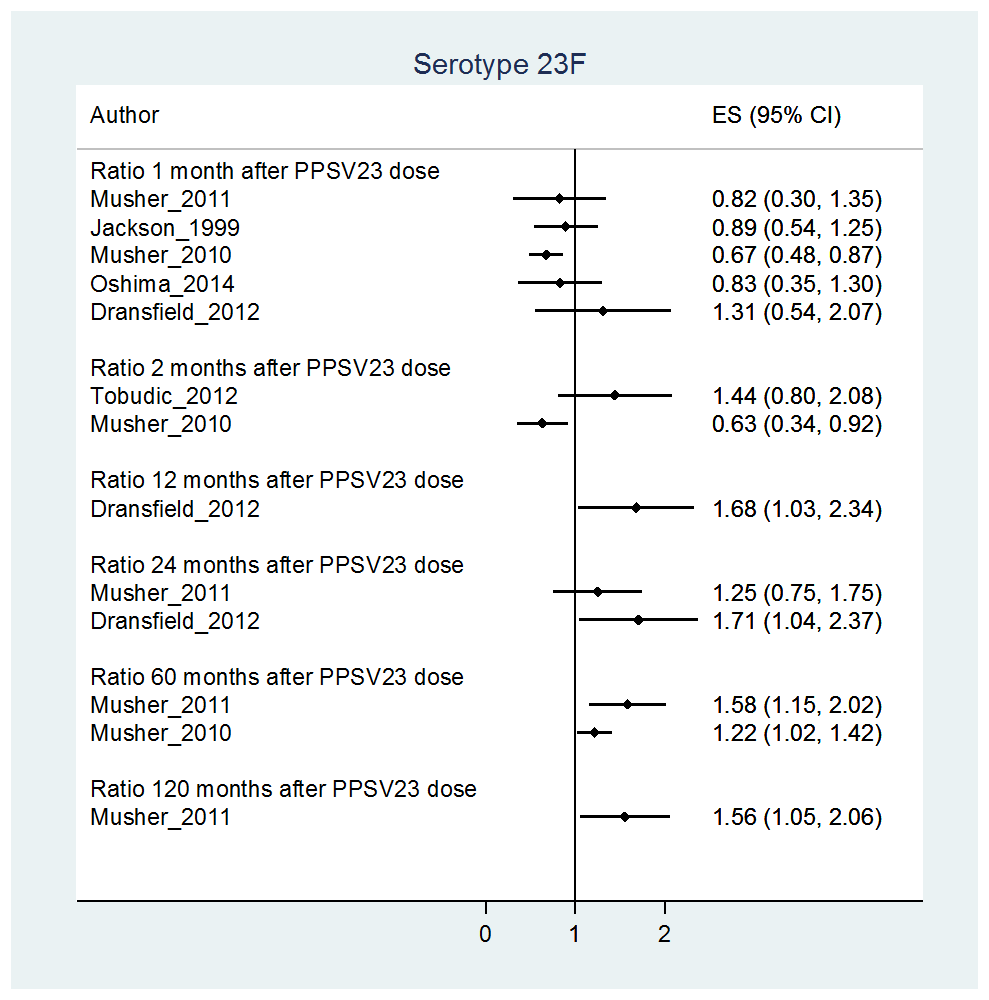


**Appendix 4**: Ratios (2^nd^ dose PPSV23/ 1^st^ dose PPSV23) of opsonophagocytic assays (OPA), by author.

**4.1. Hammitt et al., 2011**


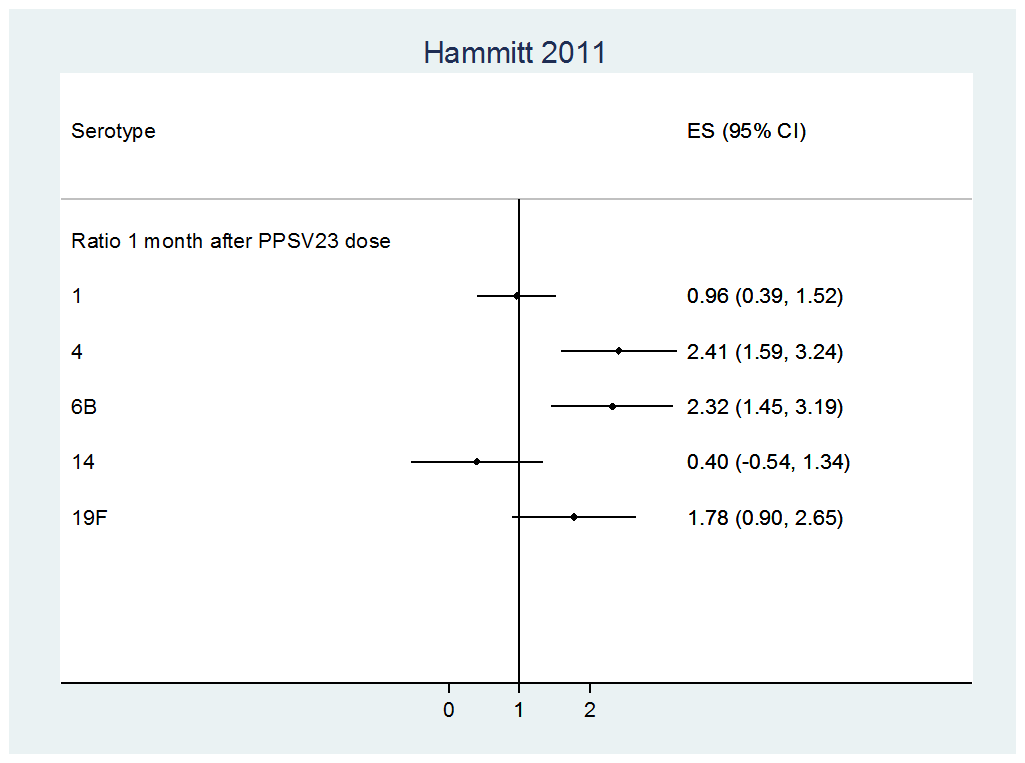


**4.2. Oshima et al., 2014**

**
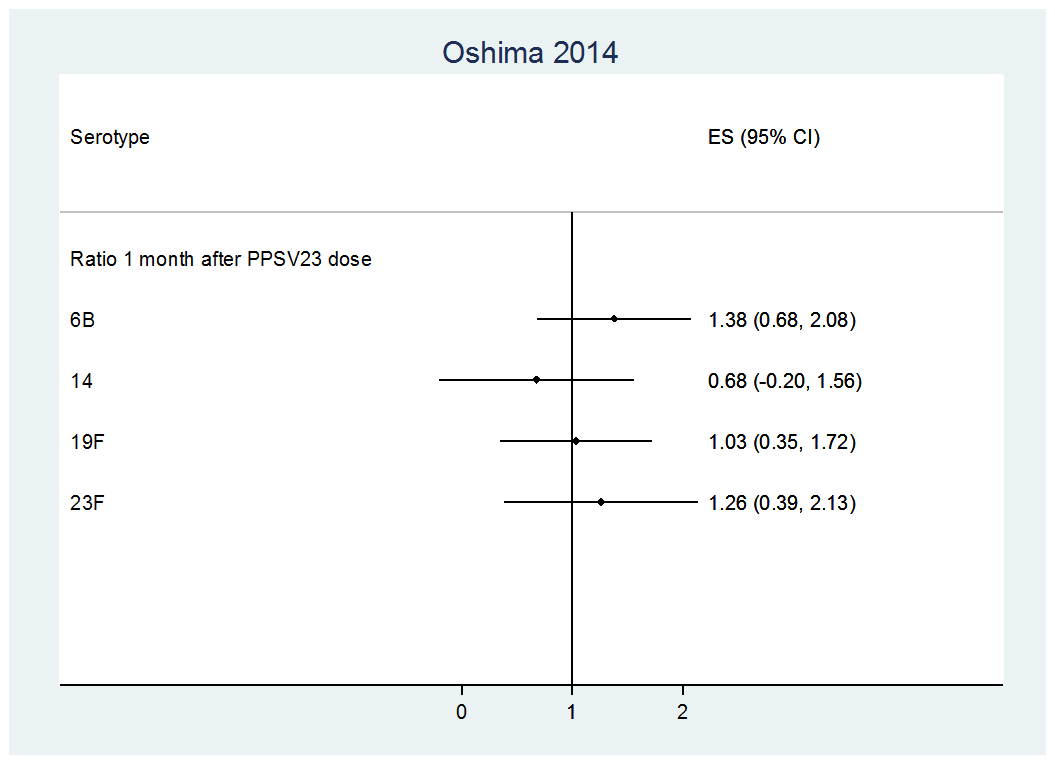
**

**References (excluded full-texts)**

1. Miernyk KM, Butler JC, Bulkow LR, Singleton RJ, Hennessy TW, Dentinger CM, Peters HV, Knutsen B, Hickel J, Parkinson AJ: **Immunogenicity and reactogenicity of pneumococcal polysaccharide and conjugate vaccines in alaska native adults 55-70 years of age**. *Clinical infectious diseases : an official publication of the Infectious Diseases Society of America* 2009, **49**(2):241-248.

2. Croxtall JD, Keating GM: **Pneumococcal polysaccharide protein D-conjugate vaccine (Synflorix; PHiD-CV)**. *Paediatric drugs* 2009, **11**(5):349-357.

3. Gilbertson DT, Guo H, Arneson TJ, Collins AJ: **The association of pneumococcal vaccination with hospitalization and mortality in hemodialysis patients**. *Nephrology, dialysis, transplantation : official publication of the European Dialysis and Transplant Association - European Renal Association* 2011, **26**(9):2934-2939.

4. Johnstone J, Eurich DT, Minhas JK, Marrie TJ, Majumdar SR: **Impact of the pneumococcal vaccine on long-term morbidity and mortality of adults at high risk for pneumonia**. *Clinical infectious diseases : an official publication of the Infectious Diseases Society of America* 2010, **51**(1):15-22.

5. Mooney JD, Weir A, McMenamin J, Ritchie LD, Macfarlane TV, Simpson CR, Ahmed S, Robertson C, Clarke SC: **The impact and effectiveness of pneumococcal vaccination in Scotland for those aged 65 and over during winter 2003/2004**. *BMC infectious diseases* 2008, **8**:53.

6. Sarmiento E, Rodriguez-Hernandez C, Rodriguez-Molina J, Fernandez-Yanez J, Palomo J, Anguita J, Perez JL, Lanio N, Fernandez-Cruz E, Carbone J: **Impaired anti-pneumococcal polysaccharide antibody production and invasive pneumococcal infection following heart transplantation**. *Int Immunopharmacol* 2006, **6**(13-14):2027-2030.

7. Brandao AP, de Oliveira TC, de Cunto Brandileone MC, Goncalves JE, Yara TI, Simonsen V: **Persistence of antibody response to pneumococcal capsular polysaccharides in vaccinated long term-care residents in Brazil**. *Vaccine* 2004, **23**(6):762-768.

8. Konradsen HB, Rasmussen C, Ejstrud P, Hansen JB: **Antibody levels against Streptococcus pneumoniae and Haemophilus influenzae type b in a population of splenectomized individuals with varying vaccination status**. *Epidemiology and infection* 1997, **119**(2):167-174.

9. Musher DM, Groover JE, Rowland JM, Watson DA, Struewing JB, Baughn RE, Mufson MA: **Antibody to capsular polysaccharides of Streptococcus pneumoniae: prevalence, persistence, and response to revaccination**. *Clinical infectious diseases : an official publication of the Infectious Diseases Society of America* 1993, **17**(1):66-73.

10. Grimfors G, Soderqvist M, Holm G, Lefvert AK, Bjorkholm M: **A longitudinal study of class and subclass antibody response to pneumococcal vaccination in splenectomized individuals with special reference to patients with Hodgkin's disease**. *European journal of haematology* 1990, **45**(2):101-108.

11. Davidson M, Bulkow LR, Grabman J, Parkinson AJ, Chamblee C, Williams WW, Lanier AP, Schiffman G: **Immunogenicity of pneumococcal revaccination in patients with chronic disease**. *Archives of internal medicine* 1994, **154**(19):2209-2214.

12. Anderson EL, Becherer PR, Belshe RB: **Revaccination with pneumococcal and hepatitis B vaccines**. *JAMA : the journal of the American Medical Association* 1993, **270**(22):2735-2736.

13. Lu CL, Chang SY, Chuang YC, Liu WC, Su CT, Su YC, Chang SF, Hung CC: **Revaccination with 7-valent pneumococcal conjugate vaccine elicits better serologic response than 23-valent pneumococcal polysaccharide vaccine in HIV-infected adult patients who have undergone primary vaccination with 23-valent pneumococcal polysaccharide vaccine in the era of combination antiretroviral therapy**. *Vaccine* 2014, **32**(9):1031-1035.

14. Crum-Cianflone NF, Huppler Hullsiek K, Roediger M, Ganesan A, Patel S, Landrum ML, Weintrob A, Agan BK, Medina S, Rahkola J *et al*: **A randomized clinical trial comparing revaccination with pneumococcal conjugate vaccine to polysaccharide vaccine among HIV-infected adults**. *The Journal of infectious diseases* 2010, **202**(7):1114-1125.

15. Iyer AS, Leggat DJ, Ohtola JA, Duggan JM, Georgescu CA, Al Rizaiza AA, Khuder SA, Khaskhely NM, Westerink J: **Response to Pneumococcal Polysaccharide Vaccination in HIV-Positive Individuals on Long Term Highly Active Antiretroviral Therapy**. *J AIDS Clin Res* 2015, **6**(2).

16. Crum-Cianflone NF, Roediger M, Huppler Hullsiek K, Ganesan A, Landrum M, Weintrob A, Agan B, Medina S, Rahkola J, Hale B *et al*: **The association of ethnicity with antibody responses to pneumococcal vaccination among adults with HIV infection**. *Vaccine* 2010, **28**(48):7583-7588.

17. Waites KB, Canupp KC, Chen YY, DeVivo MJ, Nahm MH: **Revaccination of adults with spinal cord injury using the 23-valent pneumococcal polysaccharide vaccine**. *The journal of spinal cord medicine* 2008, **31**(1):53-59.

18. Jackson LA, Neuzil KM, Nahm MH, Whitney CG, Yu O, Nelson JC, Starkovich PT, Dunstan M, Carste B, Shay DK *et al*: **Immunogenicity of varying dosages of 7-valent pneumococcal polysaccharide-protein conjugate vaccine in seniors previously vaccinated with 23-valent pneumococcal polysaccharide vaccine**. *Vaccine* 2007, **25**(20):4029-4037.

19. Jackson LA, Gurtman A, van Cleeff M, Jansen KU, Jayawardene D, Devlin C, Scott DA, Emini EA, Gruber WC, Schmoele-Thoma B: **Immunogenicity and safety of a 13-valent pneumococcal conjugate vaccine compared to a 23-valent pneumococcal polysaccharide vaccine in pneumococcal vaccine-naive adults**. *Vaccine* 2013, **31**(35):3577-3584.

20. Lackner TE, R GH, J JH, Davey C, Guay DR: **Pneumococcal polysaccharide revaccination: immunoglobulin g seroconversion, persistence, and safety in frail, chronically ill older subjects**. *Journal of the American Geriatrics Society* 2003, **51**(2):240-245.

21. Rodriguez R, Dyer PD: **Safety of pneumococcal revaccination**. *Journal of general internal medicine* 1995, **10**(9):511-512.

22. Linnemann CC, Jr., First MR, Schiffman G: **Revaccination of renal transplant and hemodialysis recipients with pneumococcal vaccine**. *Archives of internal medicine* 1986, **146**(8):1554-1556.

23. Weintrub PS, Schiffman G, Addiego JE, Jr., Matthay KK, Vichinsky E, Johnson R, Lubin B, Mentzer WC, Ammann AJ: **Long-term follow-up and booster immunization with polyvalent pneumococcal polysaccharide in patients with sickle cell anemia**. *The Journal of pediatrics* 1984, **105**(2):261-263.

24. Burwen DR, La Voie L, Braun MM, Houck P, Ball R: **Evaluating adverse events after vaccination in the Medicare population**. *Pharmacoepidemiology and drug safety* 2007, **16**(7):753-761.

25. Brown A, Rock C, Bhuachalla C, Coulter T, Dowling C, Bergin C: **Variable serological response to PPV in HIV-positive patients - A need to review pneumococcal boost-prime strategies? 14th International Congress on Infectious Diseases (ICID). International Journal of Infectious Diseases; VOL: 14; p. e451-e452 /March 2010/**. 2010.

26. Serpa JA, Valayam J, Musher DM, Rossen RD, Pirofski LA, Rodriguez-Barradas MC: **V(H)3 antibody response to immunization with pneumococcal polysaccharide vaccine in middle-aged and elderly persons**. *Clinical and vaccine immunology : CVI* 2011, **18**(3):362-366.
